# Effects of combination treatment with durvalumab plus tremelimumab on the tumor microenvironment in non-small-cell lung carcinoma

**DOI:** 10.1007/s00262-021-03065-5

**Published:** 2021-10-08

**Authors:** Li Cheng, Todd Creasy, Fernanda Pilataxi, Lydia Greenlees, Luis Vence, Sriram Sridhar, Katie Streicher

**Affiliations:** 1 Translational Science, Oncology R&D, AstraZeneca, Gaithersburg, MD USA; 2 Translational Science, BioPharmaceuticals R&D, AstraZeneca, Gaithersburg, MD USA

**Keywords:** Durvalumab, Tremelimumab, IFN-γ production, T cell proliferation and activation, EMT, Angiogenesis

## Abstract

The rapid development of immune checkpoint blockade (ICB) therapies has revolutionized the cancer treatment landscape and brightened the long-term forecast for many cancer patients. However, the specific genomic and proteomic changes in tumors treated with different ICB treatments have yet to be fully characterized. We treated four non-small-cell lung carcinoma (NSCLC) tumor digests ex vivo with the anti-PD-L1 antibody durvalumab (D) alone or in combination with the anti-CTLA-4 antibody tremelimumab (T) to explore changes in gene and protein expression associated with these ICB therapies. All four tumors showed a robust increase in interferon gamma (IFN-γ) production (100–300% higher than isotype control) in both D- and D + T-treated tumors. Three of the four tumors showed additional increases in IFN-γ production with D + T compared with D (40–70%). A substantial reduction in interleukin 10 (IL-10) was also found in three of the four tumors (reduced to 4–8%) in response to D and D + T. Conventional CD4 + /CD8 + populations and T cell activation markers increased after D and D + T treatment. D and D + T upregulated multiple IPA pathways involving T cell activation. D + T resulted in additional upregulation of Th1/Th2 pathways through a different set of genes, as well as greater reduction in genes involved in epithelial-mesenchymal transition (EMT), angiogenesis, and cancer stemness. Our results demonstrated that D + T augmented the effects of D in the microenvironment of this set of NSCLC tumors. The specific impact of D + T on the regulation of EMT, angiogenesis, and cancer stemness warrants further evaluation in a larger set of tumors.

**Supplementary Information:**

The online version contains supplementary material available at 10.1007/s00262-021-03065-5.

## Introduction

In the past decade, immunotherapies that unleash the body’s immune system against tumors have been remarkably successful in halting or shrinking even advanced tumors and prolonging patient survival [1]. The diverse field of immunotherapy encompasses the study of immune checkpoint blockade (ICB), cancer vaccines, adoptive cell therapies, and oncolytic virus therapies, among others. The largest and most well-studied category of immunotherapy drugs is ICB, which blocks inhibitory signals of T cell activation and enables tumor-reactive T cells to mount an effective antitumor response [2]. ICB therapies have shown significant clinical benefit for a minority of patients who demonstrate durable responses. Unfortunately, an unmet medical need remains for many patients whose disease does not respond to checkpoint inhibitors [1]. Thus, there is a growing need to identify predictive biomarkers that will improve the selection of patients who will best respond to ICB therapy.

ICB combination therapies are a promising strategy for improving patient responses and outcomes. In 2015, the combination of nivolumab (a monoclonal antibody against programmed cell death protein 1 [PD-1]) and ipilimumab (a monoclonal antibody against cytotoxic T-lymphocyte-associated protein 4 [CTLA-4]) became the first combination regimen to receive approval by the U.S. Food and Drug Administration (FDA) for the treatment of wild-type *BRAF V600* melanoma. Both PD-1/PD-L1 and CTLA-4 are negative signals for T cell activation, but the location, the timing and the signaling mechanisms of the inhibition are different. The combination of PD-1/PD-L1 inhibition and CTLA-4 inhibition was hypothesized to work synergistically to induce T cells to orchestrate an antitumor immune response [2]. This combination is now approved not only in wild-type BRAF V600 melanoma, but also for patients with unresectable or metastatic melanoma regardless of *BRAF* status; for previously untreated patients with intermediate- and poor-risk advanced renal cell carcinoma; and for previously treated microsatellite instability high/mismatch repair deficient metastatic colorectal cancer, where high microsatellite instability or mismatch repair deficiency served as biomarkers. Additionally, in 2020, this combination was approved for first-line treatment of adult patients with metastatic non-small-cell lung cancer (NSCLC), based on studies in which tumor PD-1 ligand (PD-L1) expression (≥ 1%) was used as a biomarker. Combinations with other PD-1/PD-L1 and CTLA-4 inhibitors have also been tested; the MYSTIC trial was a phase 3 randomized, open-label, trial comparing the anti-PDL1 antibody durvalumab (D) as monotherapy or combined with the anti-CTLA-4 antibody tremelimumab (T) with platinum-based chemotherapy as first-line treatment in patients with metastatic or locally advanced (stage IV) NSCLC. The MYSTIC trial did not meet its primary endpoint of overall survival in the randomized patient population; however, exploratory analyses identified a biomarker, blood tumor mutational burden, for which a threshold of ≥ 20 mutations per megabase was associated with optimal overall survival (OS) benefit for D + T combination treatment. In a similar clinical trial, NEPTUNE, D and T were combined as first-line treatment for patients with metastatic NSCLC; however, in this trial, the combination of D + T did not meet the primary endpoint of improving OS compared to standard-of-care chemotherapy in patients whose blood TMB was ≥ 20 mutations per megabase, as seen in MYSTIC. Finally, results from an open-label, multicenter, phase 2 trial (NCT02519348), evaluating the safety and efficacy of D and D + T in patients with advanced hepatocellular carcinoma, showed an increase in median OS when a priming dose of 300 mg of T was added to D every 4 weeks [3], and this response was associated with a unique T cell profile in tumors from patients in the T 300 mg + D arm, suggesting complementary biological activity [3]. Although some biomarkers have been previously associated with response to combination therapy with PD-1/PD-L1 and CTLA-4 inhibitors, it is still unclear which patients are most likely to respond and what key mechanisms are associated with this response.

In this study, we treated NSCLC tumor digests ex vivo with D, either alone or in combination with T, to explore the direct effects of ICB on the tumor microenvironment (TME). The results may provide insight into the mechanisms of action of ICB combinations in general and may aid efforts to characterize the effects of each of these drugs in particular. Additional insight into ICB mechanisms of action could help to identify which patients are most likely to benefit from which ICB combinations and, of particular relevance to this study, which patients will benefit more from D + T than from D alone.

## Materials and methods

### Scheme of ex vivo TIL model

**Fig. 1:**
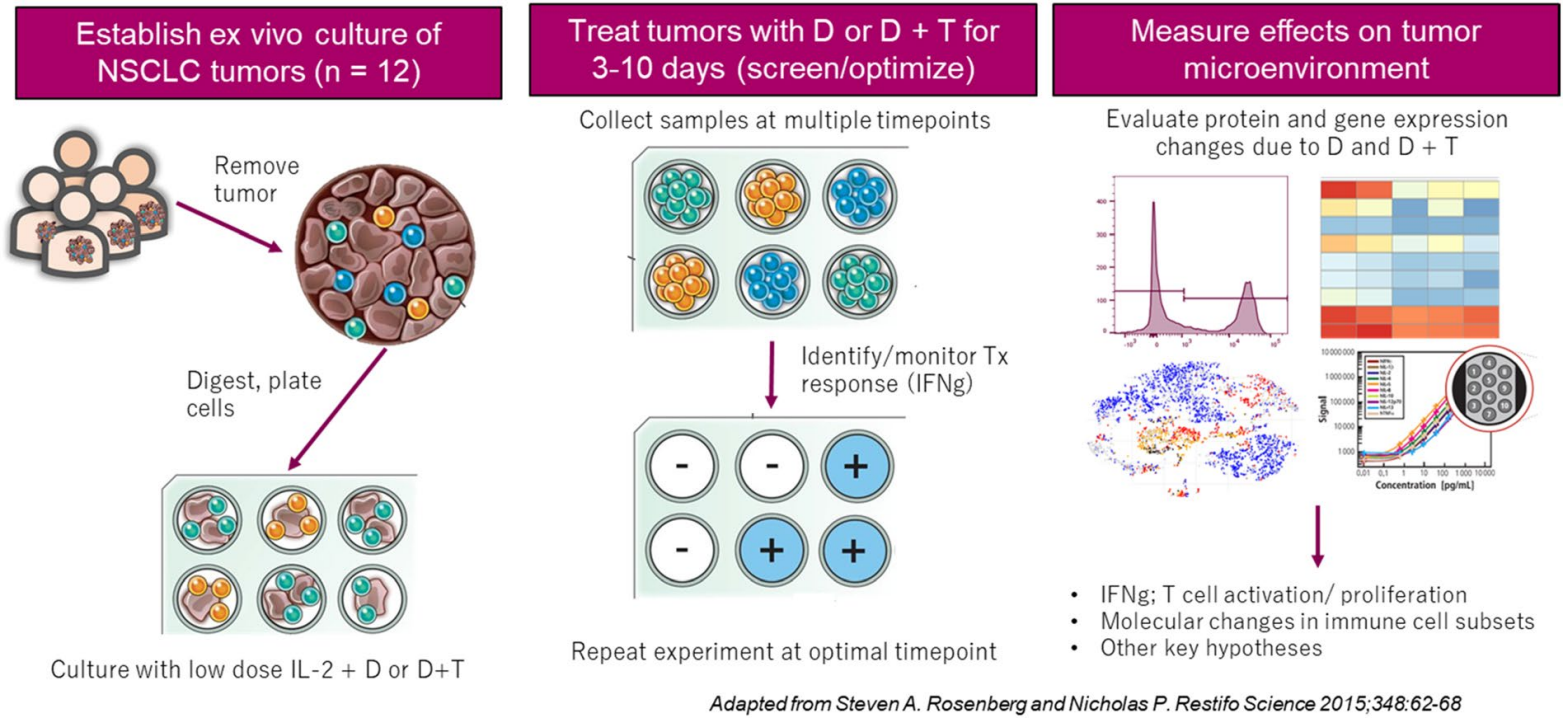
Scheme of ex vivo TIL model. Dissociated NSCLC tumor digests were cultured in medium containing low-dose IL-2 plus 20 µg/mL D, 20 µg/mL D + T, or the appropriate isotype controls. TILs were evaluated for changes in cytokine production by multiplex assay, immune-related markers by flow cytometry, and gene expression by microarray analysis

Frozen NSCLC tumor digests (untreated patients, three squamous cell carcinomas and one adenocarcinoma) were purchased from Discovery Life Sciences and cultured in 96-well plates in a medium containing low-dose interleukin 2 (IL-2) plus 20 µg/mL D alone or combined with 20 µg/mL T or the appropriate isotype controls (Fig. 1). The response of each tumor to drug was evaluated by interferon gamma (IFN-γ) expression, which was measured by using the V-PLEX Proinflammatory Panel 1 Human Kit (Meso Scale Diagnostics) at multiple time points. Experiments were repeated after identification of the responding tumors and the time points when tumors exhibited maximum response. Tumor-infiltrating lymphocytes (TILs) were evaluated for changes in cytokine production by multiplex assay (Meso Scale Diagnostics), immune-related markers by flow cytometry, and gene expression by microarray analysis.

### Dissociated tumor processing

Tumor digests were seeded at 2.5 × 10^5^ cells per well in 96-well plates containing 200 μL of complete medium and were treated with 60 U/mL IL-2 plus 20 μg/mL D, D + T, or isotype control. Complete medium consisted of RPMI 1640 medium, 10% Human Male AB Serum (Access Biologicals), 25 mmol/L HEPES (hydroxyethyl piperazineethanesulfonic acid), 100 U/mL penicillin, 100 μg/mL streptomycin, 2 mmol L-glutamine, 5.5 × 10^−5^ mol/L β-mercaptoethanol, and 10 μg/mL gentamicin. The plates were placed in a humidified incubator at 37 °C with 5% CO_2_. Supernatant was collected on days 3, 7, and 10 or 11 after treatment to measure cytokines, using assay kits from Meso Scale Diagnostics. Cells were harvested at baseline and at the end of treatment and submitted for flow cytometry or genomic studies.

### Multiplex assay

The V-PLEX Proinflammatory Panel 1 Human Kit (Meso Scale Diagnostics), which measures Interferon gamma (IFN-γ), IL-1β, IL-2, IL-4, IL-6, IL-8, IL-10, IL-12 p70, IL-13, and tumor necrosis factor alpha (TNFα), was used to quantify cytokine expression. To measure tumor growth factor-beta (TGF-β) expression level, the U-PLEX TGF-β Combo (hu) (Meso Scale Diagnostics), which measures TGF-β1, TGF-β2, and TGF-β3, was used. Plates were analyzed on an S6000 legacy imager (Meso Scale Diagnostics). Assays from Meso Scale Diagnostics were performed according to the manufacturer’s instructions. All standards and samples were measured in duplicate or triplicate.

### Flow cytometry

Tumor cells harvested at baseline or at the end of treatment were counted and normalized to the lowest total cell number. Cells were stained with the following monoclonal antibodies in two panels: Live/Dead Fixable Blue stain kit (catalog no. L23105; Thermo Fisher Scientific), APC/Cyanine7 anti-human cluster of differentiation 45 protein (CD45) (catalog no. 304014; BioLegend), BUV395 mouse anti-human CD4 (catalog no. 564724; BD Biosciences), Brilliant Violet 605 anti-CD8 (catalog no. 301040; BioLegend), PE anti-mouse/rat/human FOXP3 (catalog no. 320008; BioLegend), Alexa Fluor 488 anti-human CD127 (IL-7Rα) (catalog no. 351314; BioLegend), BV786 Mouse Anti-Human CD25 (catalog no. 563701; BD Biosciences), PerCP/Cyanine5.5 anti-human CD326 (EpCAM) (catalog no. 324214; BioLegend), Brilliant Violet 421 anti-human CD28 (catalog no. 302930; BioLegend), PE/Cyanine7 anti-human/mouse/rat CD278 (ICOS) (catalog no. 313521; BioLegend), APC anti-human CD279 (PD-1) (catalog no. 329908; BioLegend), APC anti-human IFN-γ (catalog no. 506510; BioLegend), and BV421 anti-human/mouse granzyme B (catalog no. 515408; BioLegend). Cells were stained in FACS buffer (2% fetal bovine serum in phosphate-buffered saline with 2 mM ethylenediaminetetraacetic acid) and fixed with Foxp3/Transcription Factor Staining Buffer Set (catalog no. 00-5523-00; Thermo Fisher Scientific) according to the manufacturer’s protocol. All samples were run on an LSR II flow cytometer (BD Biosciences) and analyzed with FlowJo software (BD Biosciences). Briefly, for every single flow cytometric antibody, we used an isotype control antibody to discriminate between positive and negative cells.

### Microarray experiment

Tumor cells were lysed in Buffer RLT (Qiagen) plus β-mercaptoethanol to prevent changes in gene expression profile. The lysed cells were mixed with an equal volume of 70% ethanol and then subjected to RNA isolation with the RNeasy Micro Kit (Qiagen) according to the manufacturer’s protocol. RNA concentration was determined on a Qubit Fluorometer 3.0 (Thermo Fisher Scientific), and quality was assessed on a 4200 TapeStation RNA ScreenTape (Agilent).

Generation of biotin-labeled antisense RNA amplified from 50 ng of total RNA was accomplished with the Pico WTA System V2 (Ovation) and Encore Biotin Module kits (NuGEN Technologies). Five micrograms of each biotin-labeled antisense RNA was fragmented and hybridized on Affymetrix Human Genome U133 Plus 2.0 GeneChip arrays (Thermo Fisher Scientific). All GeneChip washing, staining, and scanning procedures were performed with Affymetrix standard equipment according to the manufacturer’s protocols. Data capture and array quality assessments were performed with the GeneChip Operating Software tool (Thermo Fisher Scientific).

### Microarray data analysis

Microarray intensity values normalized by robust multiarray average were subjected to quality control measurements to assess relative log expression, normalized unscaled standard errors, signal density distribution and RNA degradation using AffyPLM (version 1.60.0) from Bioconductor (version 3.9) in R (version 3.6.0).

Differences in gene expression between treatment groups were determined by analysis of variance, adjusting for variability among the four tumors assessed. Genes exhibiting a change in expression of at least 1.4-fold (*P* ≤ 0.01) were considered to be differentially expressed, and the biological functions of these genes were further explored via pathway analysis.

Differentially expressed genes were considered for pathway and signature analyses to assess their associated biological functions. Pathways that were overrepresented among these differentially expressed genes were identified using ingenuity pathway analysis (IPA) (version 01–12; Qiagen). Immune-related gene signatures were also used to identify genes and gene sets that could be of biological interest with respect to response to immunotherapy. Heat maps were generated among samples across the four tumors to assess differences in tumor-specific expression of these gene signatures.

## Results

### Tumor microenvironment

**Fig. 2:**
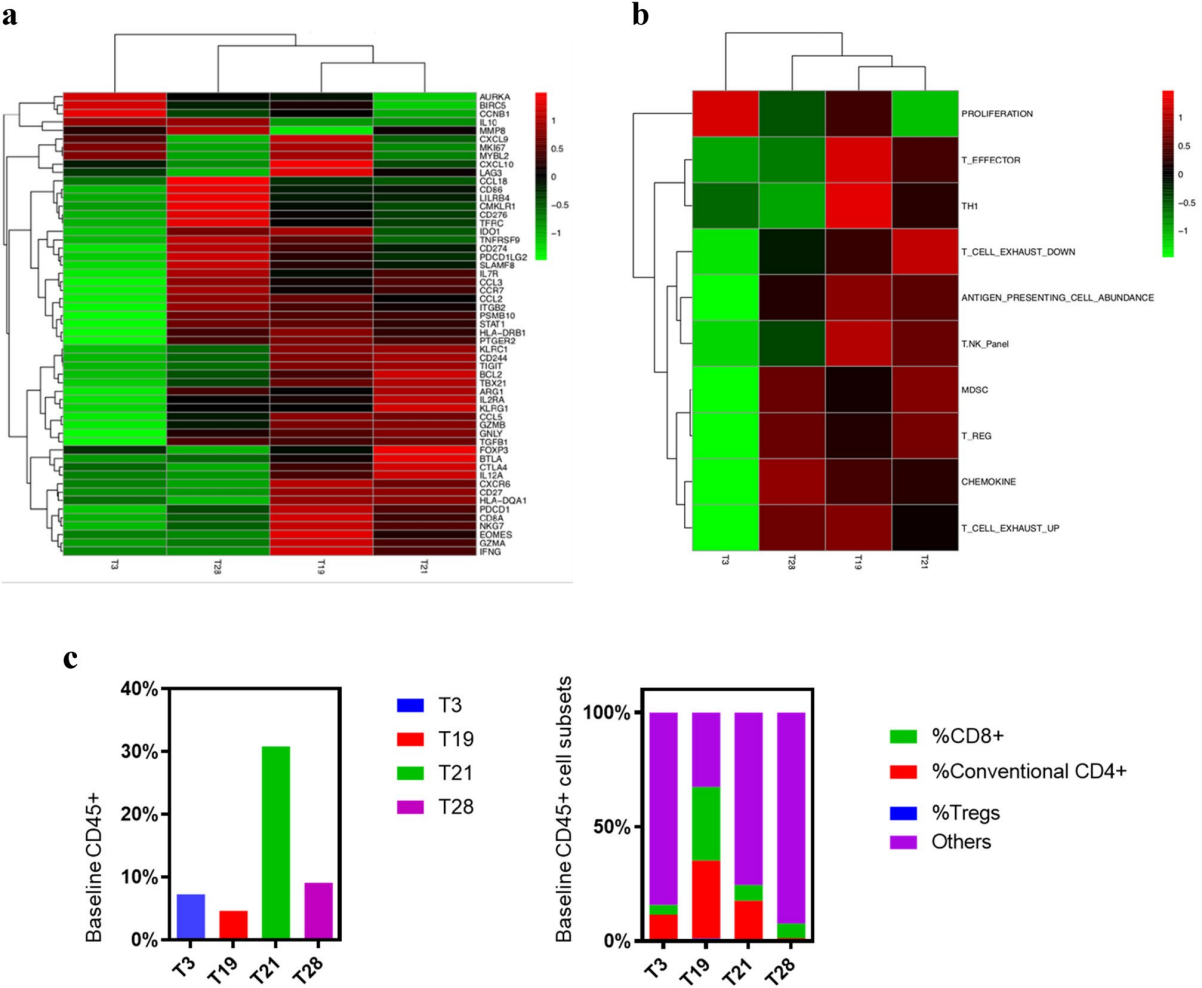
Baseline heat map of T3, T19, T21, and T28 revealed the heterogeneity the four NSCLC tumors. **a** Baseline global heat map. **b** Baseline heat map of immune-related signatures. **c** Baseline immune and CD45 + cell subsets characterized by flow cytometry

To characterize the molecular heterogeneity of the four NSCLC tumors (designated T3, T19, T21, and T28) included in our ex vivo experiments, microarray analysis was performed prior to treatment (Fig. 2a, b). The global heat map revealed the heterogeneity of the tumors. To focus on the immune contexture, we evaluated the expression of 10 gene signatures related to immune cell abundance and activation state (Fig. 2b). Interestingly, the four NSCLC tumors had some overlapping patterns but were highly heterogeneous in immune composition and activity. Based on the heat map, T19 and T21 appeared to be very similar, but T3 and T28 were notably different from the rest. For example, of the four tumors, T3 exhibited the highest proliferation activity (fold change [FC], 1.54–3.26) and the lowest myeloid-derived suppressor cell and regulatory T cell activity (FC, 0.34–0.68) of the four tumors. T19 and T21 showed higher effector T cell activity (FC, 4.01–9.80) and stronger IFN-γ and T helper cell 1 (Th1) activity (FC, 1.82–3.11) than T3 and T28.

We also measured T cell subsets in T3, T19, T21, and T28 by flow cytometry prior to ex vivo treatment (Fig. 2c). T21 had the most abundant percentage of CD45-expressing (CD45^+^) cells (30.80%) of the four tumors, followed by T28 (9.08%), T3 (7.24%), and T19 (4.61%). T19 had the highest percentage of conventional CD4^+^ cells (34.01%) in its CD45^+^ population, followed by T21 (16.84%), T3 (11.5%), and T28 (1.33%). The descending order of the tumors with percentages of CD8^+^ cells was T19 (32.2%), T21 (6.89%), T28 (6.33%) and T3 (4.18%). Overall, T21 exhibited the highest percentage of T cells (7.56%) in total cells, and T19 and T3 showed a lower percentage of T cells (3.1% and 1.14%, respectively). T28 had very few T cells at baseline level and had the lowest percentage of T cells in total cells (0.70%).

### Increased production of IFN-γ, IL-12 p70, and TNF-α and decreased production of IL-10 and TGF-β2 after D and D + T treatment

**Fig. 3:**
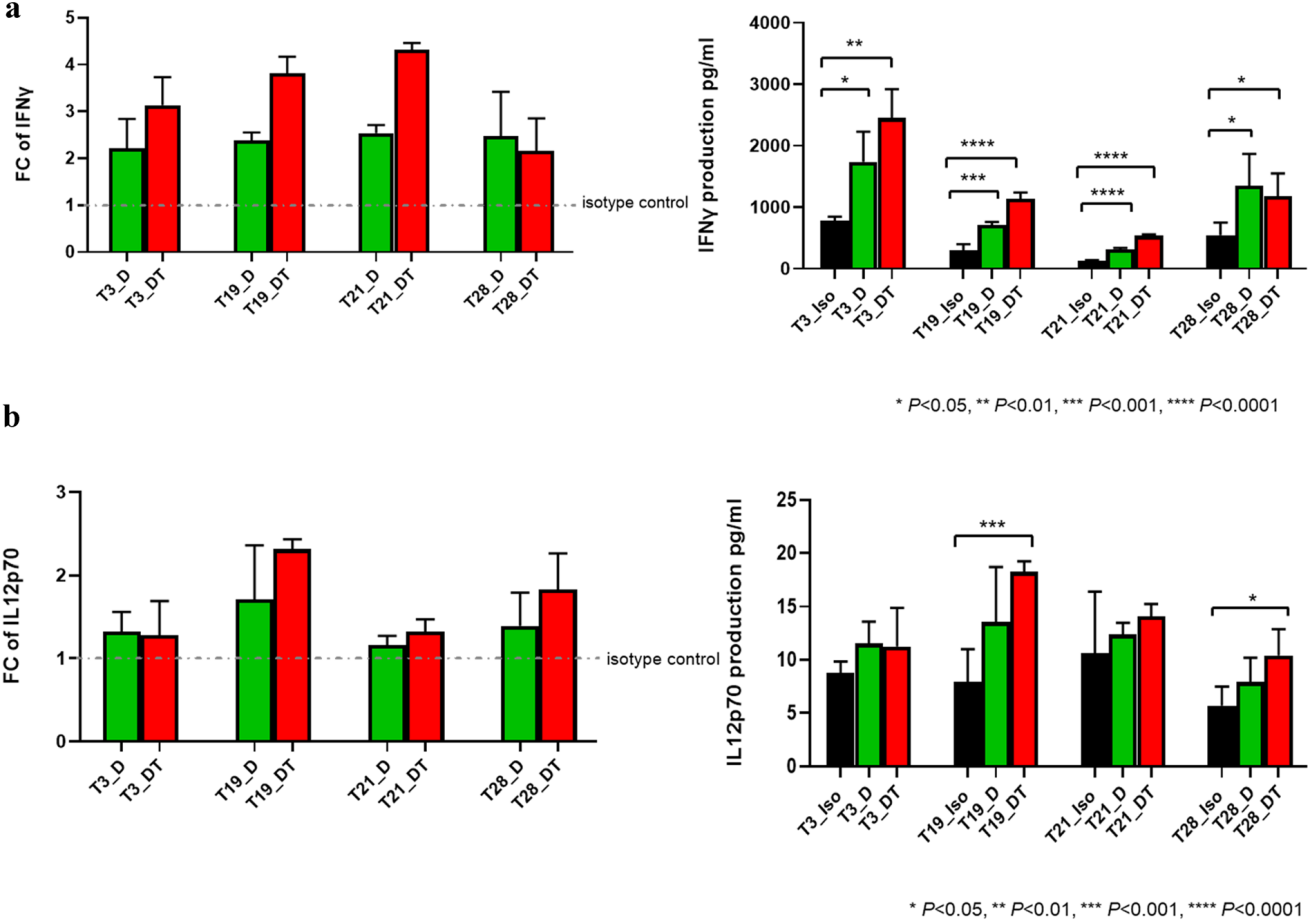

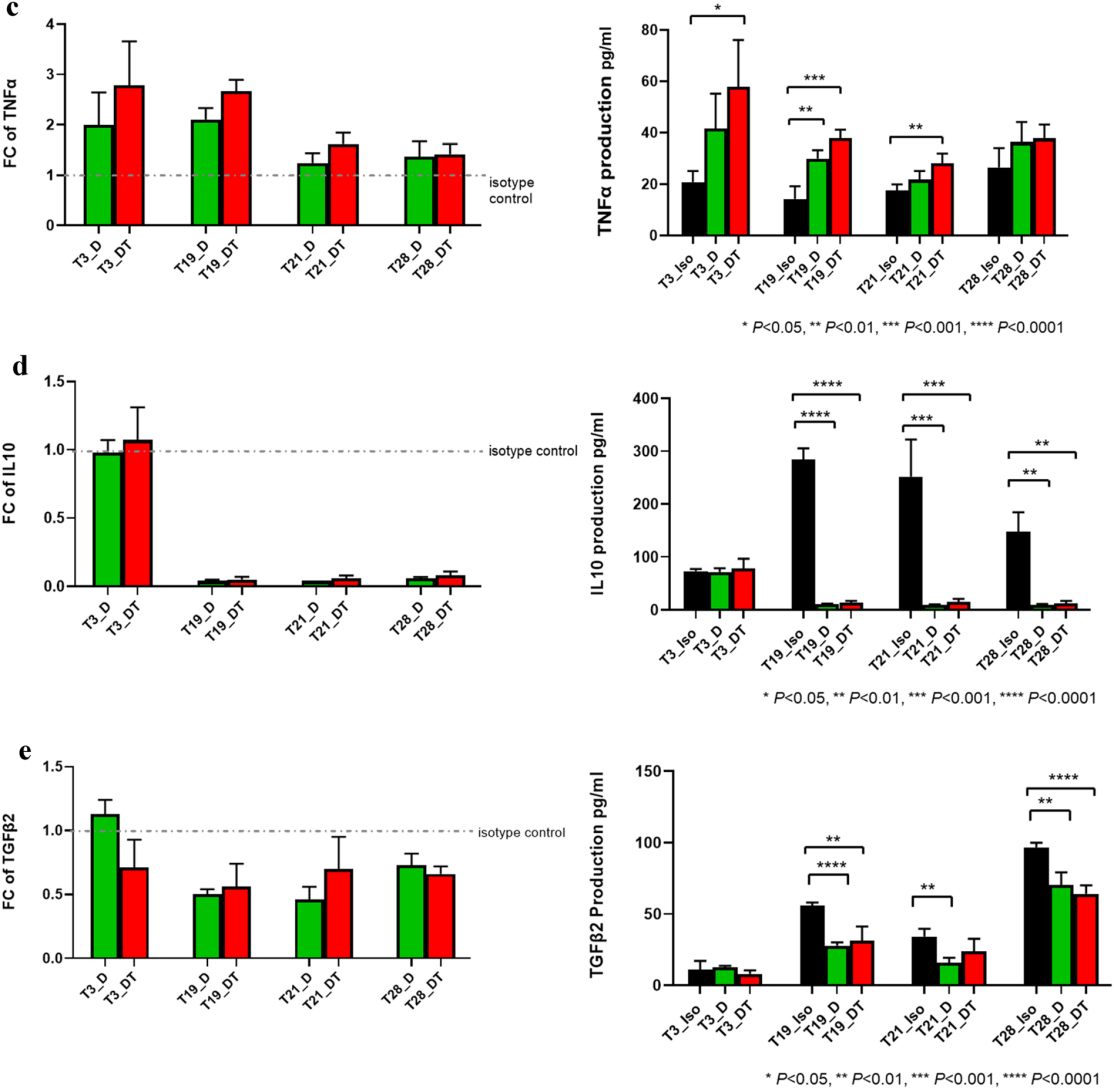
Changes in cytokine production upon treatment with D or D + T. IFN-γ, IL-12 p70, TNF-α, IL-10, and TGF-β2 production was measured by MSD multiplex assay. FC for D and D + T treatment was calculated versus individual isotype control at the time point when each tumor exhibited maximum response (i.e., the optimal time point). Raw data on the production of each cytokine at the optimal time point after treatment with D and D + T are shown: **a** increased IFN-γ production; **b** increased IL-12 p70 production; **c** increased TNF-α production; **d** decreased IL-10 production; **e** decreased TGF-β2 production

IFN-γ is a cytokine that plays a pivotal role in antitumor host immunity by inducing Th1 polarization, cytotoxic T-lymphocyte activation, and dendritic cell tumoricidal activity [4]. Therefore, we used IFN-γ production as an indicator of response to D and D + T treatment. T3, T19, T21, and T28 tumors were treated with D, D + T, or isotype control for 11 days. IFN-γ production in T3, T19, T21, and T28 reached its maximum compared with control on days 11, 7, 7, and 10, respectively (Fig. 3a; Supplement Fig. 2). When D was compared with isotype control, the descending order of the tumors with percentage increase in IFN-γ was T21 (154%, *P* < 0.0001), T28 (147%, *P* < 0.05), T19 (138%, *P* < 0.001), and T3 (121%, *P* < 0.05). When D + T was compared with isotype control, the descending order of the tumors with percentage increase in IFN-γ was T21 (332%, *P* < 0.0001), T19 (282%, *P* < 0.0001), T3 (213%, *P* < 0.01), and T28 (116%, *P* < 0.05). T28 produced equivalent amounts of IFN-γ in response to D and D + T. For T3, T19, and T21, D + T augmented the effects of D in elevating IFN-γ, the highest being T21 (D + T 70% higher than D, *P* < 0.0001), followed by T19 (D + T 61% higher than D, *P* < 0.001) and T3 (D + T 42% higher than D) (Fig. 3a).

We also examined other cytokines (IL-1β, IL-2, IL-4, IL-6, IL-8, IL-10, IL-12 p70, IL-13, and TNF-α) known to be immune active or immune suppressive by MSD multiplex assay. The main antitumor actions of IL-12 include increasing the production of IFN-γ and stimulating the growth and cytotoxicity of activated natural killer, CD8^+^, and CD4^+^ T cells [5]. At the time point when each tumor exhibited maximum response, treatment with D and D + T resulted in higher IL-12 p70 production by all four tumors (T3, 32% and 28%; T19, 71% and 131% [*P* < 0.001]; T21, 16% and 32%; T28, 39% and 83% [*P* < 0.05], respectively) (Fig. 3b). D + T resulted in higher production of IL-12 p70 than D alone in T19 (35%), T28 (32%), and T21 (14%). D and D + T treatment also resulted in higher TNF-α production by all four tumors (T3, 99% and 178% [*P* < 0.05]; T19, 110% [*P* < 0.01] and 166% [*P* < 0.001]; T21, 24% and 62% [*P* < 0.01]; T28, 37% and 42%, respectively) (Fig. 3C). D + T resulted in higher production of TNF-α than D alone for T3 (40%), T21 (31%; *P* < 0.05), and T19 (27%; *P* < 0.05).

IL-10 has been widely accepted to be an immunosuppressive cytokine in cancer, as it plays important roles in the promotion of tumor immune escape [6]. In our study, T19, T21, and T28 all showed substantial reduction in IL-10 production (reduced to 4–8%, *P* < 0.01) in response to D and D + T (Fig. 3d). T3 produced the lowest level of IL10 as isotype control comparing to the other three tumors, which may explain why no decrease in IL-10 production was seen with D or D + T. Another well-known immunosuppressive cytokine is TGF-β, which promotes tumor growth by inhibiting T cell proliferation, activation, and effector functions [7]. We tested all three isoforms of TGF-β—TGF-β1, TGF-β2, and TGF-β3—and found that only TGF-β2 displayed a meaningful and consistent change over isotype control. T3, T19, T21, and T28 all showed a moderate reduction in TGF-β2 production (reduced by 27%–54%) in response to D and D + T (Fig. 3e), and similar reductions in response to D were shown by all except T3, which maintained the same level of TGF-β2 as isotype control when treated with D. T3 expressed a very low level of TGF-β2 compared with isotype control, which might account for the lack of response of this tumor to D.

### Upregulation of conventional CD4^+^ and CD8^+^ populations and T cell activation markers after D and D + T treatment

**Fig. 4:**
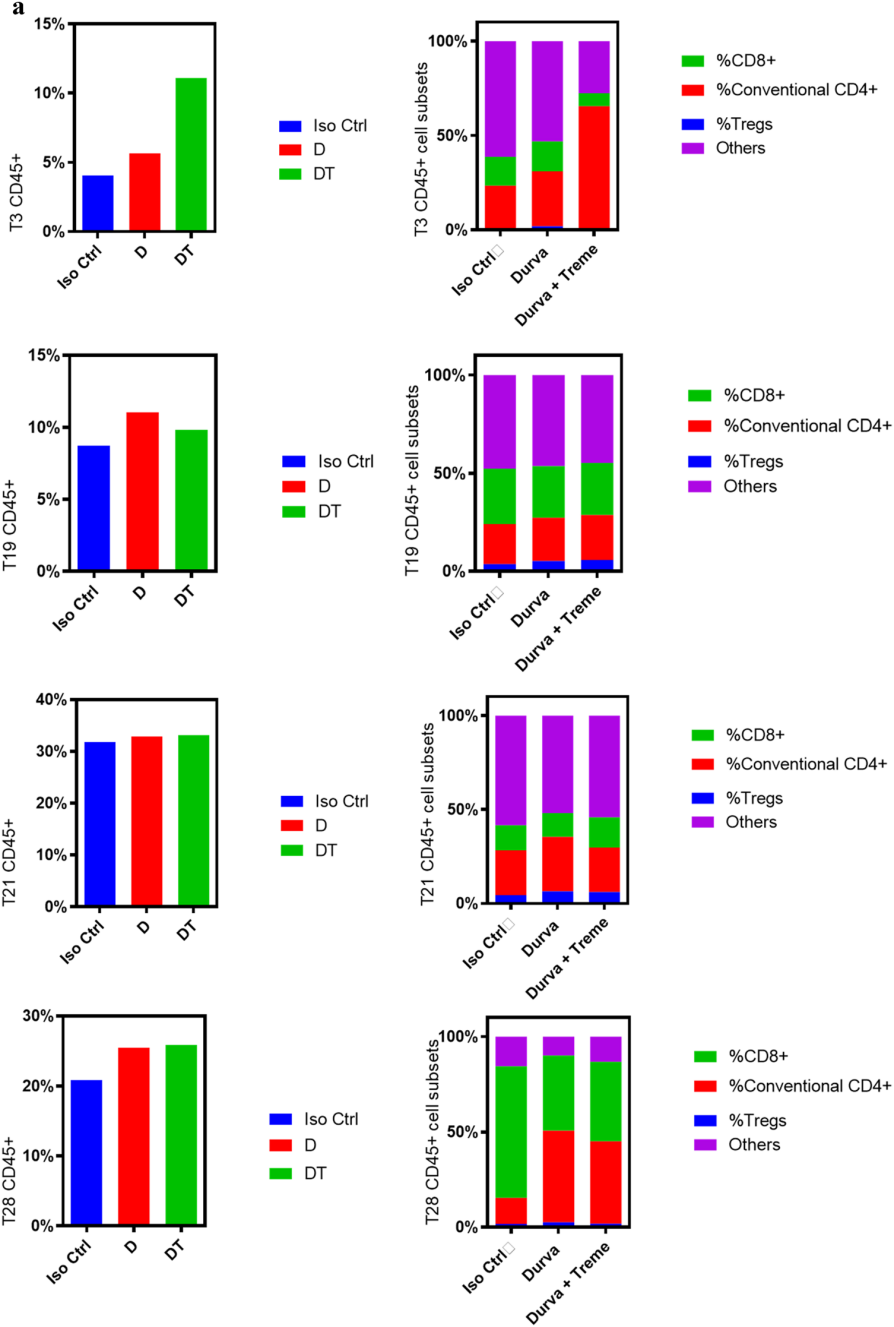

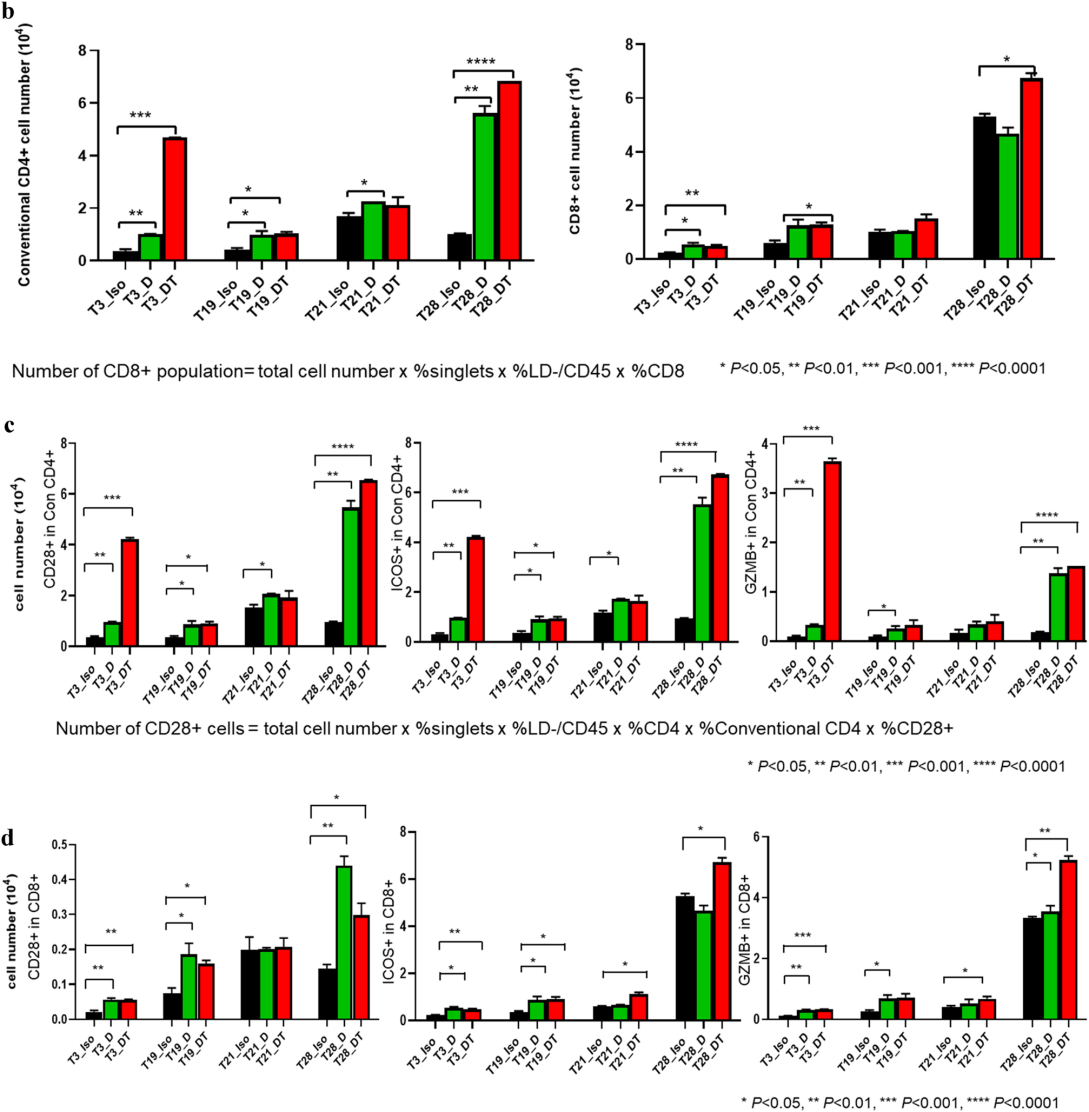
T cell proliferation and increased expression of T cell activation markers with D and D + T treatment. **a** Immune and CD45 + cell subsets were characterized by flow cytometry after treatment. **b** Conventional CD4^+^ and CD8^+^ cells were calculated based on total cell counts and conventional CD4^+^/CD8^+^ percentages obtained from flow cytometry. **c** Cell numbers of CD28^+^, ICOS^+^, and GZMB^+^ in conventional CD4^+^ and CD8^+^ populations were calculated based on total cell counts and percentage of each T cell activation marker as determined by flow cytometry

Flow cytometry data indicated that the four tumors showed different levels of increases in conventional CD4^+^ and CD8^+^ cells after treatment with D or D + T (Fig. 4b). These increases were due to an increase in the percentage of both CD45^+^ and conventional CD4^+^/CD8^+^ cells (Fig. 4a). Increases in conventional CD4^+^ cells after D and D + T treatment were highest in T28, which had levels that were 4.48-fold (*P* < 0.01) and 5.69-fold (*P* < 0.0001) higher, respectively, than for isotype control, followed by T3, which had 1.72-fold (*P* < 0.01) and 11.61-fold (*P* < 0.001) higher levels; T19, which had 1.34-fold (*P* < 0.05) and 1.45-fold (*P* < 0.05) higher levels; and T21, which had 34% (*P* < 0.05) and 25% higher levels than isotype control when treated with D and D + T, respectively (Fig. 4b). T3 showed an additional 3.64-fold (*P* < 0.0001) increase in conventional CD4^+^ cells when treated with D + T compared with D alone, making it the best responder to T among the four tumors. T28 also showed an additional 27% (*P* < 0.05) increase in conventional CD4^+^ cells when treated with D + T compared with D alone. Ratio of Treg/conventional CD4 + decreased significantly to 43% (*P* < 0.01) and 30% (*P* < 0.01) in response to D and D + T, respectively, for T28 (supplement Fig. S1), which was the only one among the four tumors showing significant reduction in Treg/conventional CD4 + . Changes in CD8^+^ cells in response to D and D + T treatment were relatively similar, in the range of 88–222%, among the four tumors (Fig. 4b).

To evaluate T cell activity and cytotoxicity, we measured CD28, inducible costimulator (ICOS), and granzyme B (GZMB) levels in CD4^+^ and CD8^+^ T cell subsets after D and D + T treatment. We observed an increase in CD28^+^, ICOS^+^, and GZMB^+^ cells in conventional CD4^+^ T cells in all tumors after both D and D + T treatment, as measured by flow cytometry (Fig. 4c). T28 showed the greatest increase in CD28^+^, ICOS^+^, and GZMB^+^ cells in conventional CD4^+^ population, with 4.67–6.50-fold (*P* < 0.01) higher levels after D treatment and 5.80–7.28-fold (*P* < 0.0001) higher levels after D + T treatment. In T3, CD28^+^, ICOS^+^, and GZMB^+^ cells in conventional CD4^+^ population were 1.83–2.78-fold (*P* < 0.01) higher after D treatment and 11.33–40.45-fold (*P* < 0.0001) higher after D + T treatment than in isotype control. T3 exhibited a 5.19–13.55-fold (*P* < 0.001) increase in CD28^+^, ICOS^+^, and GZMB^+^ cells in conventional CD4^+^ population in response to D + T treatment compared with D alone. The changes in CD28^+^, ICOS^+^, and GZMB^+^ cells in the CD8^+^ population were not as robust or consistent as in the CD4^+^ population, as only T3 demonstrated a clear difference (0.98–1.91-fold increase; *P* < 0.05) in all three markers in the CD8^+^ population after D and D + T treatment (Fig. 4c).

### Upregulation of T cells and downregulation of angiogenesis, EMT, and cancer stemness after D and D + T treatment

**Fig. 5:**
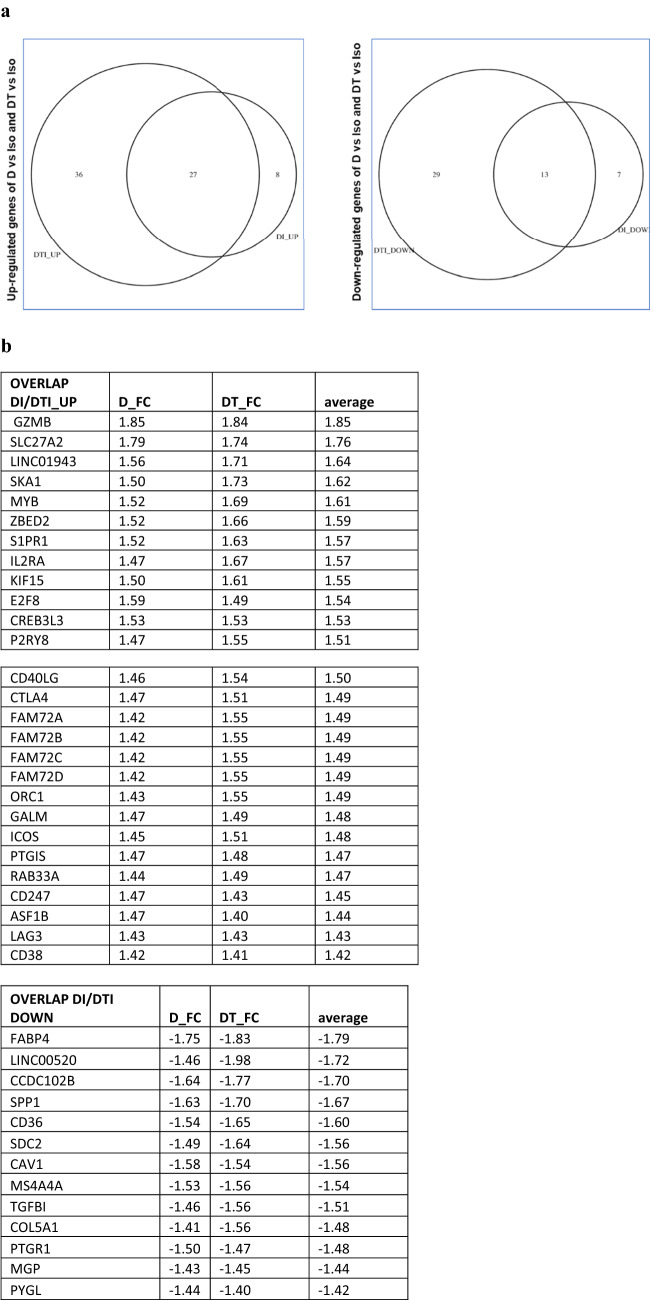

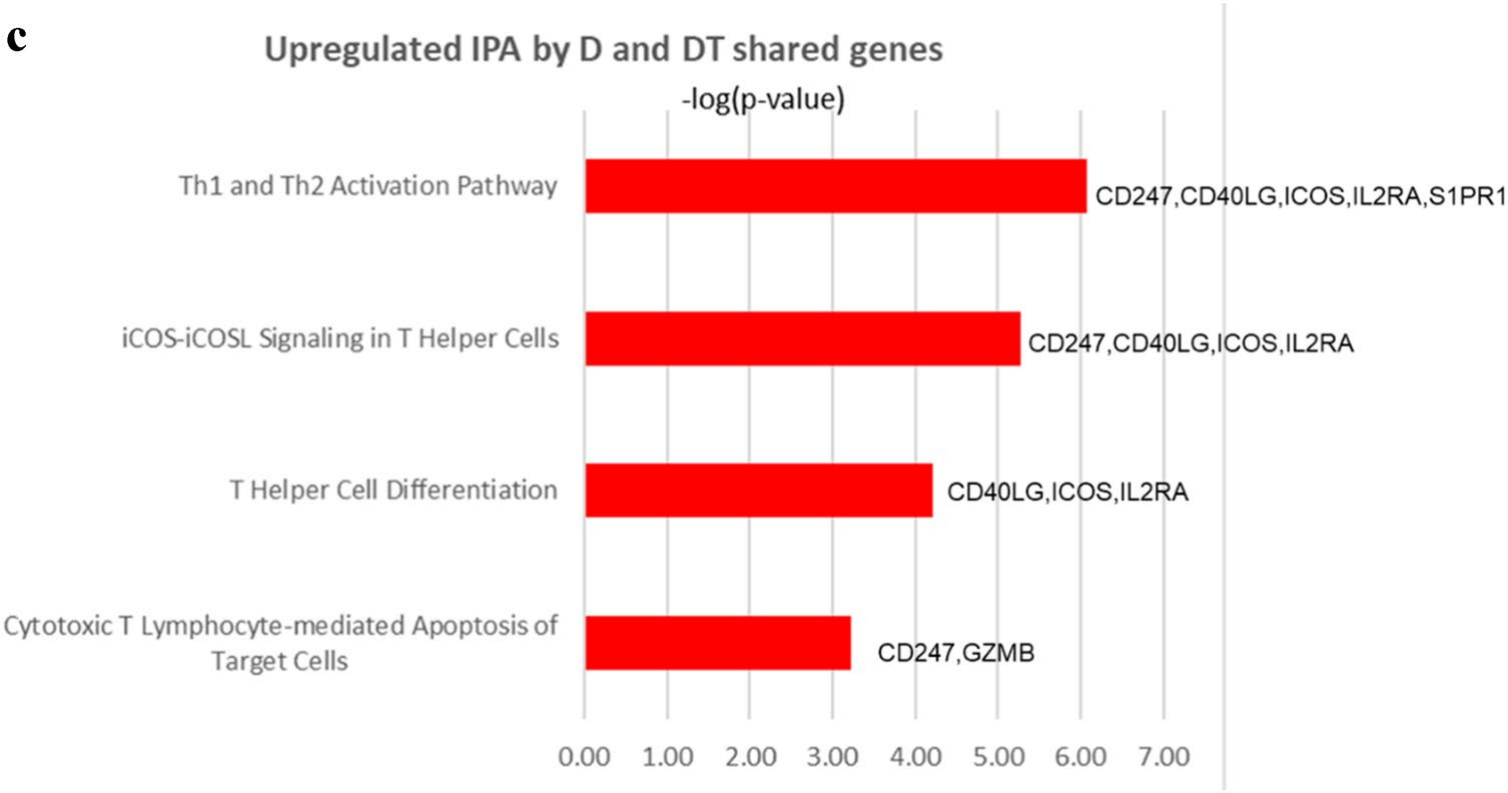

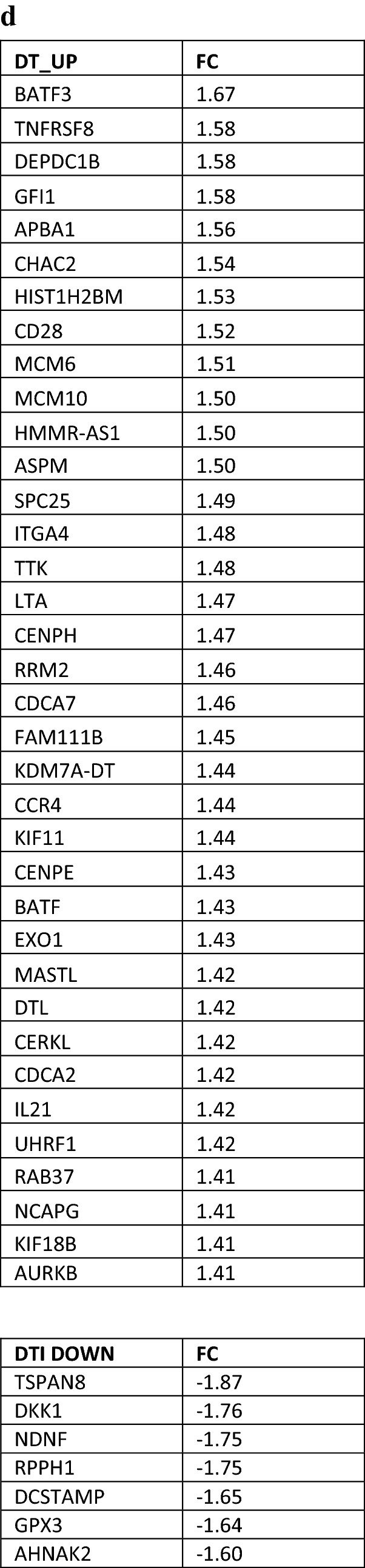

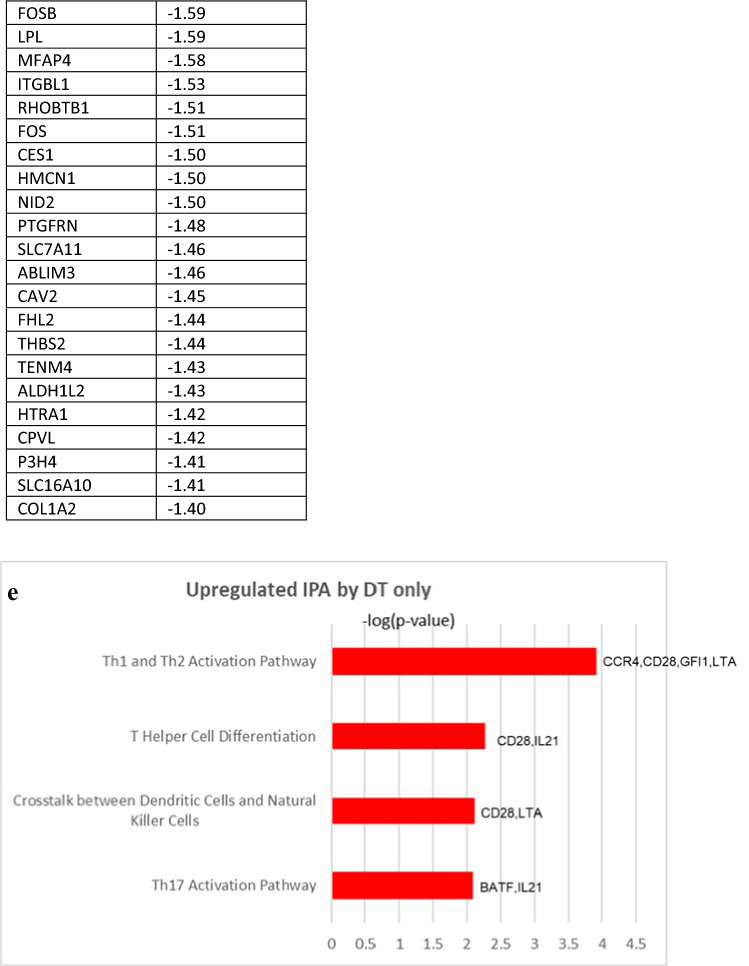
Microarray data analysis. **a** Upregulated and downregulated genes after treatment of four tumors with D and D + T. **b** Overlapping genes shared by the four tumors treated with D and D + T. Shown is the FC resulting from D compared with isotype control, D + T compared with isotype control, and average of the two. **c** Upregulated IPA pathways shared by the four tumors treated with D and D + T and − log *P* value and genes involved in the IPA pathways. **d** Unique genes of the four tumors treated with D + T. Shown is the FC in tumors treated with D + T compared with isotype control. **e** Microarray data analysis of upregulated IPA pathways in the four tumors treated with D + T and − log *P* values for genes involved in the IPA pathways

To more fully explore the effect of D and D + T treatment, we used microarray analysis to interrogate whole-transcriptome data at the time point when IFN-γ production was at its maximum. Differences in gene expression between treatment groups were determined by analysis of variance, adjusting for variability among the four tumors. A total of 120 genes were altered in both the D- and the D + T-treated tumors. Although this number was somewhat low, we considered it important to identify consistent changes across tumors, independent of immune cell diversity before treatment. Of the 120 altered genes, 40 genes were shared by the four tumors treated with D and D + T; 65 genes were unique to tumors treated with D + T, and 15 genes were unique to tumors treated with D (Fig. 5a). IPA revealed that multiple molecular pathways were altered identically and significantly (− log *P* > 1.3) with D and D + T treatment, and the following pathways were closely relevant to immune activation/regulation in cancer: (1) Th1 and Th2 activation pathway (*CD247*, *CD40LG*, *ICOS*, *IL2RA* and *S1PR1*; < FC 1.51, − log(*P* value) = 6.07 >); (2) iCOS-iCOSL signaling in T helper cells (*CD247*, *CD40LG*, *ICOS* and *IL2RA*; < FC 1.50, − log(*P* value) = 6.07 >); (3) T helper cell differentiation (*CD40LG*, *ICOS* and *IL2RA*; < FC 1.52, − log(*P* value) = 4.21 >); and (4) Cytotoxic T-lymphocyte-mediated apoptosis of target cells (*CD247*, *GZMB*; < FC 1.65, − log(*P* value) = 3.23 >) (5b). *SLC27A2* (solute carrier family 27 member 2; FC 1.76) was also upregulated by the four tumors and played an important role in maintaining lung cancer cells that were sensitive to cisplatin. No consistent pathway-level changes were found in the downregulated genes shared by tumors treated with D and D + T when analyzed with IPA. The four genes displaying the highest FC among downregulated genes were *FABP4*, *LINC00520*, *CCDC102B*, and *SPP1* (5B). *FAB4* (fatty acid binding protein 4; FC, − 1.79) and *SPP1* (secreted phosphoprotein 1, also called osteopontin; FC, − 1.67) have previously been shown to increase the metastatic potential of cancer cells, elevate angiogenesis, promote epithelial-mesenchymal transition (EMT), and suppress T cell activation in cancers [8, 9], whereas little is known about the functions of *LINC00520* and *CCDC102B* in cancer.

In addition to shared gene between D and D + T, D + T also showed some unique molecular pathway changes. Based on the IPA results, D + T treatment resulted in significant upregulation (− log *P* > 1.3) of the following pathways through a different set of genes than those regulated by D alone (Fig. 5c): (1) Th1 and Th2 activation pathway (*CCR4*, *CD28*, *GFI1*, *LTA*; < FC 1.50, − log(*P* value) = 3.92 >); and (2) T helper cell differentiation (*CD28*, *IL21*; < FC 1.47, − log(*P* value) = 2.27 >), through a different set of genes than regulated by D alone (Fig. 5c). Two other IPA pathways, crosstalk between DC and NK cells (*CD28*, *LTA*; < FC 1.50, − log(*P* value) = 2.11 >) and Th17 activation pathway (*BATF*, *IL21*; < FC 1.43, − log(*P* value) = 2.09 >), were also upregulated by D + T. We did not find any consistent downregulated pathways with D + T compared with D alone. The four genes showing the highest FC among the downregulated genes were *TSPAN8*, *DKK1*, *NDNF*, and *RPPH1* (Fig. 5c). *TSPAN8* (tetraspanin 8; FC − 1.87), *DKK1* (dickkopf‐1; FC − 1.76), *NDNF* (neuron-derived neurotrophic factor; FC − 1.75) and *RPPH1* (ribonuclease P RNA component H1; FC − 1.75) have been shown to promote cancer cell stemness, elevate angiogenesis, increase EMT, inhibit Th1 polarization, and suppress IFN-γ secretion in cancers [10–13], suggesting that D + T may have a broader effect on different types of biological pathways than D alone.

## Discussion

Our study applied an ex vivo TIL assay to investigate the molecular and genomic changes driven by D and D + T across four NSCLC tumors. From MSD multiplex assay and flow cytometry results, we observed increased production of the inflammatory cytokines IFN-γ, IL-12, and TNF-α; decreased production of other inhibitory cytokines, such as IL-10 and TGF-β2; and T cell proliferation and upregulation of T cell activation markers, all of which has been previously associated with antitumor immunity [2, 14, 15]. Microarray analysis revealed that multiple IPA pathways were altered identically in response to D and D + T treatment, despite the fact that the baseline TME looked markedly different for the four NSCLC tumors. These shared IPA pathways were closely related to T cell activation, which was downstream of the immuno-oncologic agents D and T. Our model also revealed shared gene changes regulating EMT and angiogenesis, the inhibition of which is important in order to deprive a tumor of nutrients and oxygen, to stop cancer cell metastasis, to reduce the suppression of cancer-fighting activity of cytotoxic T cells that traffic into the tumor, and to enhance immunotherapy.

Treatment of tumors with D + T also resulted in unique changes to some molecular pathways in addition to those shared with D-treated tumors. D + T upregulated IPA pathways associated with T cell activation through a different set of genes: *CD28*, *GFI1*, *IL21*, *LTA*, *BATF* (basic leucine zipper ATF-like transcription factor), and *CCR4*. Of these, *IL21* and *BATF* both induced the differentiation of Th17 cells, which have been reported to play a complex and controversial role in tumor immunity. However, several reports have described Th17 cells being able to induce the regression of established tumors and to reduce the number of tumor foci when transferred into mice [16–18]. Although the mechanism by which Th17 cells cause tumor regression is not fully understood, one study has shown that Th17 cells drove antitumor immune responses by recruiting immune cells into tumors, activating CD8^+^ effector T cells, or by directly converting Th17 cells to a Th1 phenotype and producing IFN-γ [19]. Lymphotoxin-alpha (LTA) is a member of the TNF family and is crucial for the optimal functioning of DCs [20]. DCs, which are widely accepted as the most potent antigen-presenting cells, play important roles in activating cytotoxic T cells to mount an antitumor response to ICB immunotherapy. *TSPAN8*, the most downregulated gene, promotes cancer cell stemness via activation of sonic hedgehog signaling, and its expression correlates with a poor prognosis [13]. Cancer stem cells are strongly associated with treatment resistance and tumor progression. *DKK1*, one of the top four downregulated genes in our study, has been found to signal to myeloid-derived suppressor cells in order to promote tumor growth in murine syngeneic models and to signal to CD4^+^ T cells to inhibit Th1 polarization and suppress the secretion of IFN-γ [10], and *RPPH1*, another downregulated gene, promotes EMT and macrophage M2 polarization in colorectal cancer [11]. Both myeloid-derived suppressor cells and M2 macrophages are crucial to maintain the immunosuppressive TME, protect cancer cells from the patient’s immune system, and make the tumor resistant to immunotherapy. All of the above unique gene changes in tumors treated with D + T suggest that D + T may have a more diverse and expansive impact on multiple biological pathways than D alone [21].

While these results are very interesting, there are limitations of the ex vivo model system used in this manuscript. This model was utilized in order to evaluate the effects of D and D + T on the NSCLC tumor microenvironment and more specifically, on the immune cell component of the TME, as the ex vivo system with low-dose IL-2 is optimal for promoting immune cell survival compared with other cells present in the TME. Additionally, this model was a closed system and did not permit peripheral immune cell infiltration, so we were unable to evaluate this aspect of the biological effect of D and D + T.

With encouraging observations on inflammatory cytokines, T cell activation, and gene changes regulating cancer stemness, EMT, and angiogenesis in this pilot study, we plan to expand this assay in a larger cohort to confirm our findings. The current ex vivo model does allow for full MOA to be addressed that would be possible in clinical setting, so it will be important to incorporate the results presented here into a more complex ex vivo model and/or to test hypotheses in clinical samples.

In summary, treatment of tumors with D + T augmented the effect of D in elevating IFN-γ, promoting T cell proliferation, and increasing the expression of T cell activation markers. D + T treatment resulted in additional upregulation of the Th1/Th2 pathway, Th17 pathway activation, and greater reduction in genes involved in EMT, angiogenesis, and cancer stemness. Understanding the mechanism of action of D + T may be important for generating hypotheses and designing experiments that lead to more promising biomarker development.

### Supplementary Information

Below is the link to the electronic supplementary material.

Supplementary file1 (DOCX 83 kb)

## Data Availability

The data used to support the findings of this study are available from the corresponding author upon request.

## Abbreviations

CD: Cluster of differentiation
CD40LG: CD40 ligand
CTLA-4: Cytotoxic T-lymphocyte-associated protein 4
D: Durvalumab
DC: Dendritic cell
D + T: Durvalumab plus tremelimumab
EMT: Epithelial-mesenchymal transition
FC: Fold change
FDA: U.S. Food and Drug Administration
GZMB: Granzyme B
ICB: Immune checkpoint blockade
ICOS: Inducible costimulator
IFN: Interferon
IL: Interleukin
IPA: Ingenuity pathway analysis
LTA: Lymphotoxin-alpha
NSCLC: Non-small-cell lung cancer
PD-1: Programmed cell death protein 1
PD-L1: Programmed cell death protein ligand 1
T: Tremelimumab
TGF: Tumor growth factor
Th: T helper (cell)
TIL: Tumor-infiltrating lymphocyte
TME: Tumor microenvironment
TNF: Tumor necrosis factor
