# Supplementary figures and images for: Effects of combination treatment with durvalumab plus tremelimumab on the tumor microenvironment in non-small-cell lung carcinoma
Source: Cancer Immunol Immunother. 2021 Oct 8;71(5):1167–81. doi: 10.1007/s00262-021-03065-5 (PMC9015996; doi:10.1007/s00262-021-03065-5)

**Supplementary Fig 1**


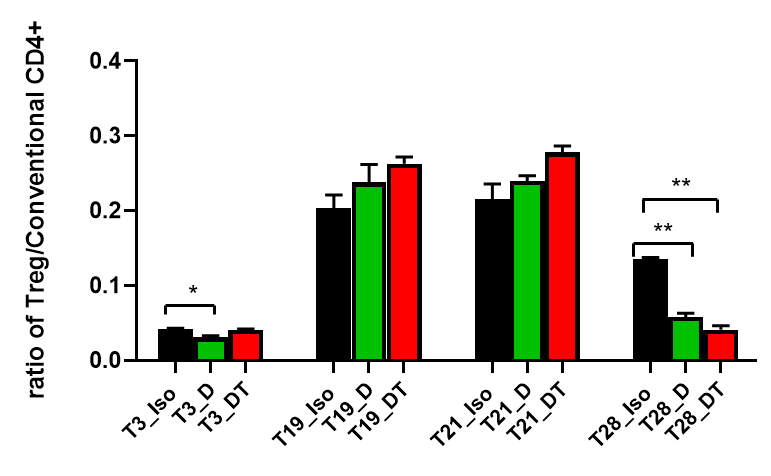


**Supplementary Fig 2**


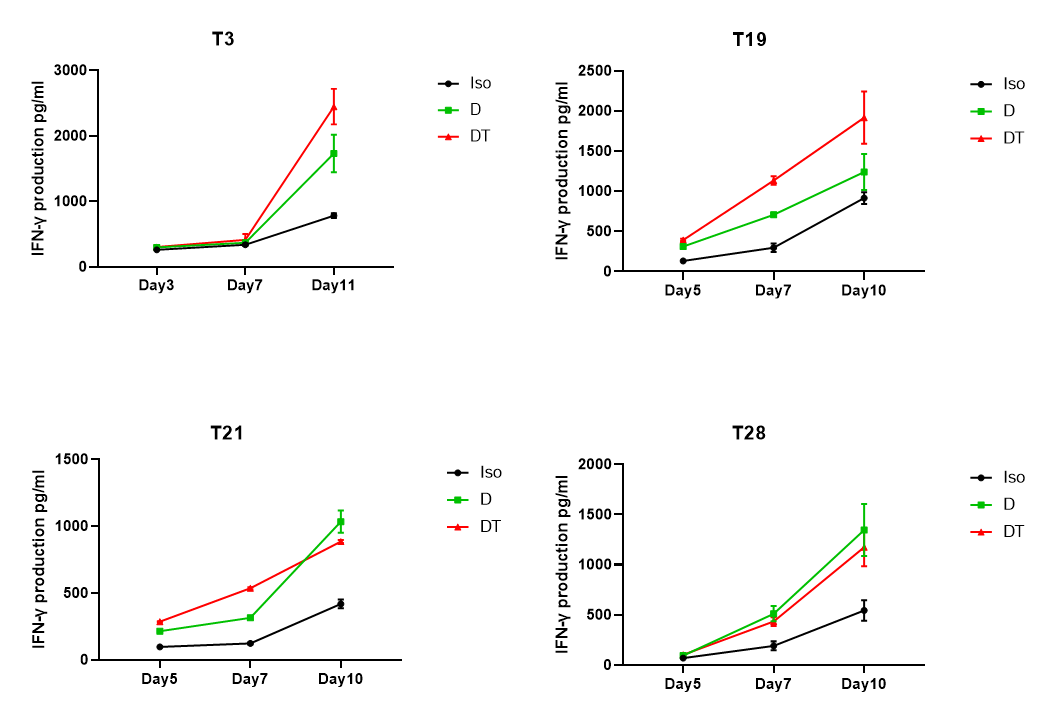


**Supplementary Fig 3**


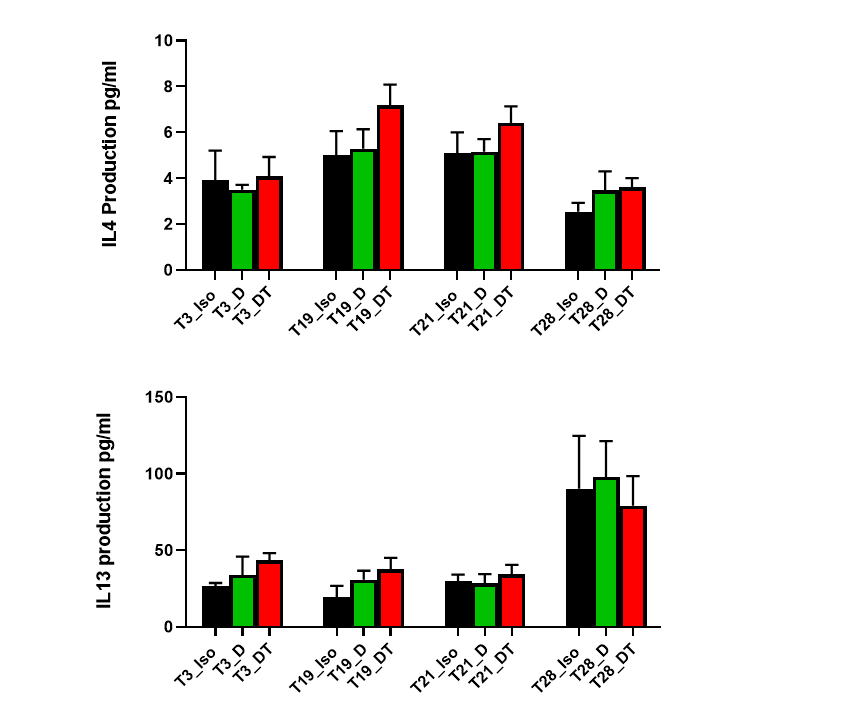

Supplement: Supplementary file 1 — Supplementary file1 (DOCX 83 kb) [file 262_2021_3065_MOESM1_ESM.docx]
